# The analysis and application of granular backfill material to reduce surface subsidence in China’s northwest coal mining area

**DOI:** 10.1371/journal.pone.0201112

**Published:** 2018-07-23

**Authors:** Erhu Bai, Wenbing Guo, Yi Tan, Daming Yang

**Affiliations:** 1 School of Energy Science and Engineering, Henan Polytechnic University, Jiaozuo, Henan, P.R. China; 2 Synergism Innovative Centre of Coal Safety Production in Henan Province, Jiaozuo, Henan, P.R. China; University of Science and Technology Beijing, CHINA

## Abstract

In China’s northwest coal mining area, the excavation of shallow buried thick coal seams has caused serious damage to the phreatic water layer and induced deterioration of the ecological environment. Backfilling is a basic method of controlling the loss of groundwater and reducing surface subsidence. In order to reduce the porosity of the backfill material and control the compression ratio of the backfill body, the grain gradation of the local aeolian sand was studied based on the geological conditions of the shallow buried coal seam in the Yulin mining area, Shaanxi province. Subsequently, aeolian sand was selected as the backfilling aggregate, and tests were implemented. The optimum proportion and slurry concentration of the backfill material were then obtained. The engineering application shows that the strength and stability of the backfill body based on the close packing theory can satisfy the requirements of supporting the overlying strata, and the integrity of overburden strata is competent. The maximum accumulated surface subsidence was measured to be 38mm, indicating that the aeolian sand-based backfill material in shallow and thick underground coal seam mining is able to protect the eco-environment and control the geo-environmental hazards, which are critical for the sustainable development of the mining industry and economic growth.

## Introduction

With the depletion of coal resources in central and eastern China, the exploitation of coal resources has also been adjusted and the policy of strategic transformation to western China has been implemented. This is consistent with China’s West Development Program and the concept of the “Silk Road Economic Belt” [1]. Therefore, the mining and production capacities of coal mines have been increasing intensively [2]. The production capacity of new large coal mines generally reaches 10 million tons per annum. The longwall panel length reaches 300 m in the dip direction (the maximum is 450 m), the mining height reaches 5 m (the maximum is 8.8 m), and the advancing speed reaches 10 m/d (the maximum is 20 m/d). According to the status of high productivity mining in northwest China, the annual production capacity of a single longwall panel is the sum of a dozen longwall panels in an eastern coal mine. However, due to the frangible eco-environment and natural conditions, stable and sustainable production cannot be assured in the arid and semiarid regions in northwest China. During the extraction process, the overburden strata would gradually sink and collapse, forming caving, fracture and bending zones above the coal seam [3–4]. For shallow buried thick coal seam mining, the overburden strata generally form only a “two-zone” model (caving zone and fracture zone), which leads to the destruction of underground aquifers and the decrease of soil fertility, nutrient supply, and biodiversity. In addition, the waste rock produced by driving entries and coal washing is typically stockpiled close to a mine site, resulting in non-productive land and posing potential threats to air and water quality. Theoretical research and field measurements show that a large scale and high-intensity underground coal mine can easily result in geo-environmental hazards, such as water and soil loss, vegetation deterioration and land desertification [5–8]. Therefore, new approaches to address this challenge are urgently needed.

In recent years, backfill coal mining has been increasingly applied in China, which has obvious advantages in terms of protecting ecological environments and controlling strata movement. Most practical engineering cases of backfill mining use gangues and waste rocks, as these mining areas are coal resource-exhausted mines in eastern China. This method can both effectively control overburden strata deformation and dispose of the waste rock [9–11]. Due to the pollutants contained in the backfill material, heavy metal pollution can result in groundwater pollution lasting decades, with the pollution intensity increasing over time [12]. At the same time, Bian et al. (2012) have already noted the potential secondary pollution in underground water quality and the underground environment caused by solid backfill technology with gangue or waste rock [13]. If the backfilling result is effective and the underground aquifers are virtually unaffected by coal mining, then this material can be used to minimize the pollution of underground water [14]. Thus, it is necessary to determine whether the waste material mixture is suitable for backfilling according to the environmental impact assessment.

Considering the special geological conditions in northwest China, groundwater is the most important water resource. While the abundant aeolian sand on the surface provides ideal anti-deformation capabilities and can be used as backfill material to fill the gob area, only bending deformation occurs in the overburden strata. Consequently, surface subsidence can be effectively controlled. Moreover, it is consistent with stable and sustainable production and ecological environmental protection. Therefore, it is necessary to study the new and pollution-free backfill materials.

This paper is organized as follows: firstly, we present a theoretical basis for the granular backfill material. This is followed by a grain gradation test of the aeolian sand. Next, the results of the property experiments for the backfill material are presented and discussed. Finally, we analyse and discuss the engineering application perspective and present our conclusions. The results indicate that backfill coal mining with loess and aeolian sand as the backfill material can effectively control local surface subsidence and help protect the environment in eco-environmental frangible areas. The goal of this study is to promote further research regarding environmental protection in northwest China.

## Theoretical basis for packing density of granular backfill material

Many scholars have studied crushed particle compaction to examine the physical and mechanical performances of backfill materials. Pappas and Mark (1993) used the Salamon and Terzaghi equations to describe the stress-strain properties and obtained the relationship between stress and the tangent and secant modulus [15]. Xu et al. (2011) studied the deformation characteristics of the backfill body and concluded that the deformation process was divided into stages of rapid, slow and stable deformation [16]. Su et al. (2012) studied the compaction characteristics of broken rock in the coal seam roof from the aspects of rock strength, block diameter, compaction stress, and the effect of the water-saturated state on the compaction property of crushed stone from the coal seam roof [17]. Zhou et al. (2016) studied the compressive deformation and energy dissipation of gangue in the loading process under conditions of different particle sizes, loading rates, and first-time stress loads [18]. Zhang et al. (2014) performed compaction experiments on loose gangues and determined the relationship between strain, expansion coefficient, and compactness, as well as some relevant characteristics relating to the compaction time [19]. After that, he investigated the fractal characteristics of compacted gangue by using compaction experiments under various stresses and different particle sizes [20]. Zhang et al (2016) defined the qualitative relationship between the mass and compensation ratios, analyzed the key factors impacting the mass ratio, and proposed the mass ratio control principles and guaranteeing measures [21]. Although the abovementioned scholars conducted experimental research on broken rock compaction, the research and application of the pollution-free granular materials used for backfilling based on the close packing theory has not been investigated, including aspects such as the grain gradation of aeolian sand, the optimum proportion, and slurry concentration.

**Fig 1:**
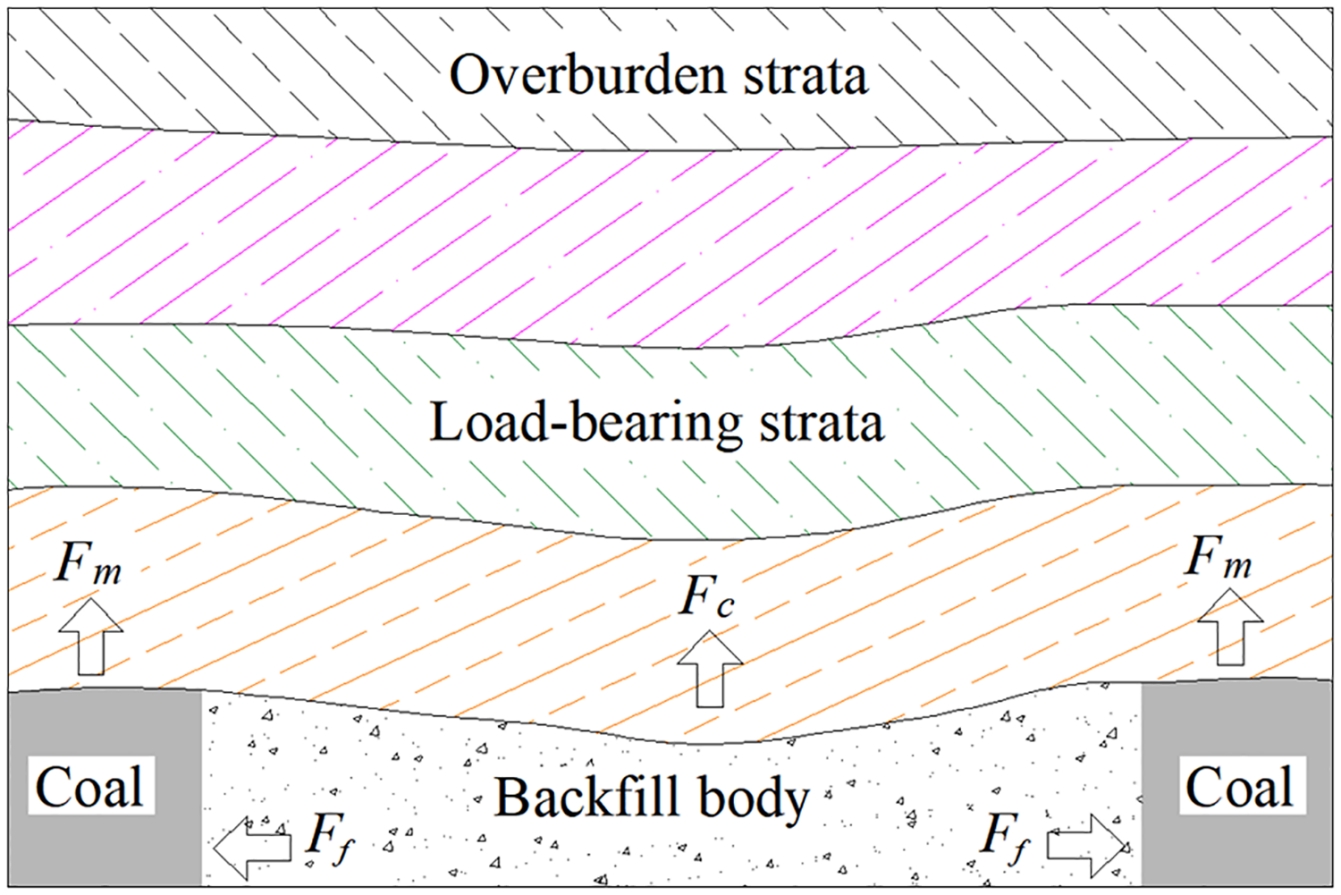
Support system with backfill body, coal pillar, and load-bearing strata.

Based on the above analysis, goaf and its components should be studied. For the backfilling mining method, when the mine-out area is filled with backfill body, the stability of overburden strata is maintained by the backfill body, coal pillar, and load-bearing strata, as shown in Fig 1. *F*_*f*_ is the supporting pressure of the backfill body on the coal pillar, *F*_*m*_ is the supporting force of the coal pillar on the overburden strata, and *F*_*c*_ is the supporting force of the backfill body on the overburden strata. Once the backfill body has been destroyed, the coal pillar stability will be influenced, and large deformation of the overburden strata will occur, resulting in large ground surface subsidence.

As seen in Fig 1, the subsidence degree of the entire load-bearing strata determines the moving range of the overburden strata, while the load-bearing strata formation is directly controlled by the stability of the backfill body and coal pillar. Therefore, the backfill rate and the compression of the backfill body may cause subsidence of the load-bearing strata. This subsidence mainly includes two parts: the unfilled height of the mine-out area (backfill rate) and the compression of the backfill body (compaction and elastic-plastic deformation of backfill body).

In order to facilitate the analysis, the ratio of the filling height to the mine-out height is regarded as the backfill rate. Namely,

$$\eta =\frac{H_{f}}{H_{g}}\times 100\%$$ (1)

Where *η* is the backfill rate (%), *H*_*f*_ is the filling height (m), and *H*_*g*_ is the goaf height (m). Then the unfilled height of the goaf area can be obtained as follows:

$$H_{u}=(1-\eta )H_{g}$$ (2)

Where *H*_*u*_ is the unfilled height of the goaf area (m).

In addition, the porosity of the backfill body is used to indicate the degree of compaction. According to the principles of soil mechanics, the compression value of the backfill body can be obtained by the following equation:

$$S_{1}=\frac{e_{0}-e}{1+e}H_{f}$$ (3)

Where *S*_1_ is the compression value produced by the backfill body, *e*_0_ is the initial porosity of the backfill body, and *e* is the porosity ratio after compaction.

According to the above formula, the porosity ratio of the backfill body needs to be further studied at a guaranteed backfill rate, and the close packing theory is used to analyze the void in the backfill material.

The theoretical basis for close packing is filling voids with smaller grains until the voids in the solid particles reach a minimum. The packing density is the ratio of the solid particle volume to the total volume, including the voids between solid particles, usually expressed by a decimal. As an important physical parameter of the solid particle system, the packing density reflects the compactness of the solid particles. In addition, another important parameter in close packing theory and a factor that improves the strength is the work performance and durability of the composite materials.

**Fig 2:**
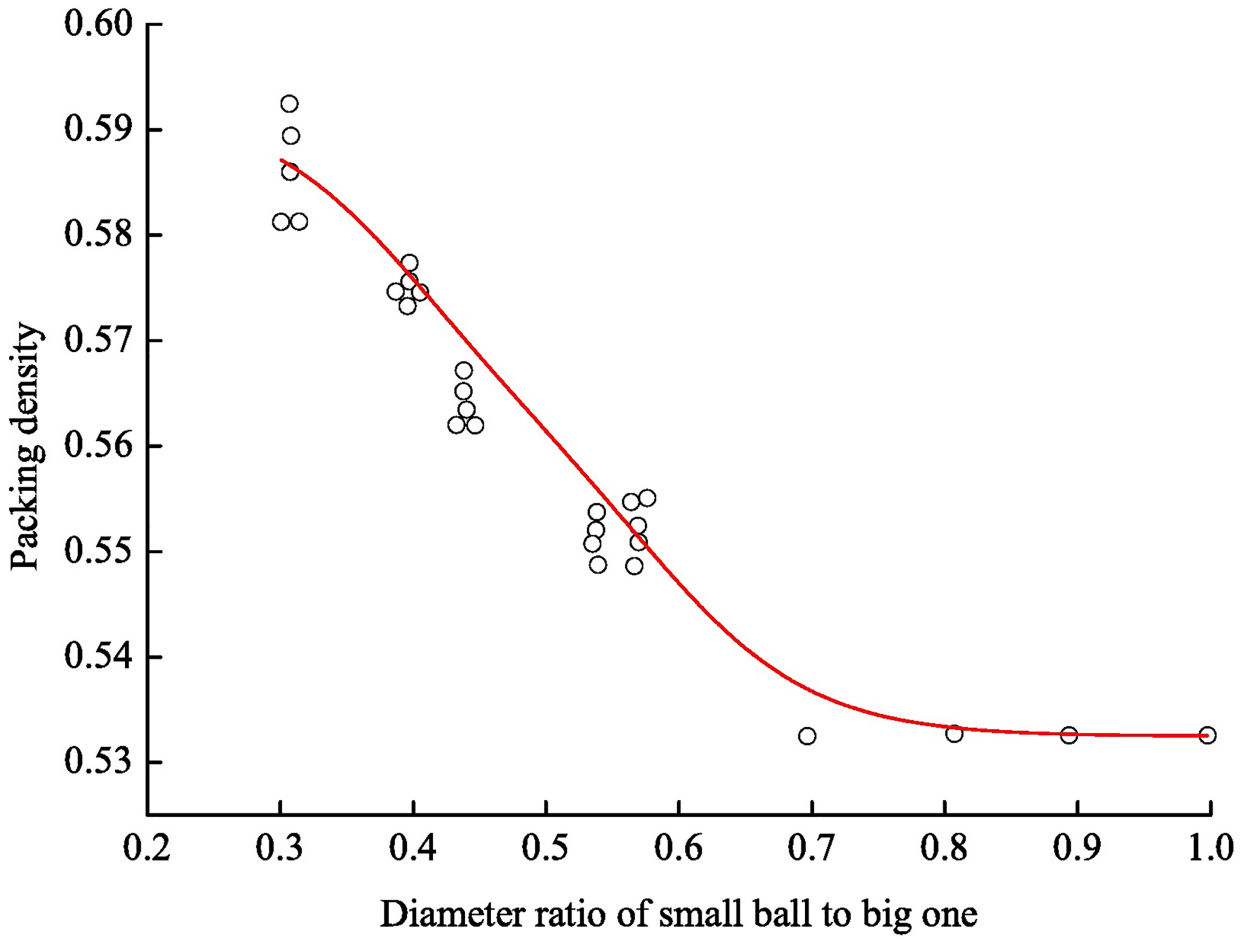
Variation of packing density with the diameter ratio of unequal spheres.

For backfill coal mining, we replace the support roof of the coal body in the original space with the backfill body and constrain the overburden strata movement to a certain extent. Then the mining effects on the surface structures can be reduced. However, the control level depends on the compactness of the backfill body, i.e. the packing density of the backfill material. According to the study on the mix packing of a binary sphere system [22], the packing density of a mixture is larger than the diameter of a sphere when the radius ratio of the mixture to an equal sphere is less than 0.7. The smaller the ratio is, the larger the packing density will be, as shown in Fig 2.

The basic viewpoints of close packing theory for the non-solid backfill method is to squeeze out the void solution to the maximum extent, retain the proper amount of water, and maximize the use of the Van Der Waals force. Therefore, the strength of the backfill body can be improved by reducing the porosity of the backfill material and increasing the friction and cohesiveness. Then, this method can be used for controlling the overlying strata movement and reducing the surface structure deformation. In the northwest coal mining area, abundant natural aeolian sand material is available on the surface, and studying the microstructure and grain gradation of aeolian sand provides a basis for backfill coal mining.

## Grain gradation test of aeolian sand

**Fig 3:**
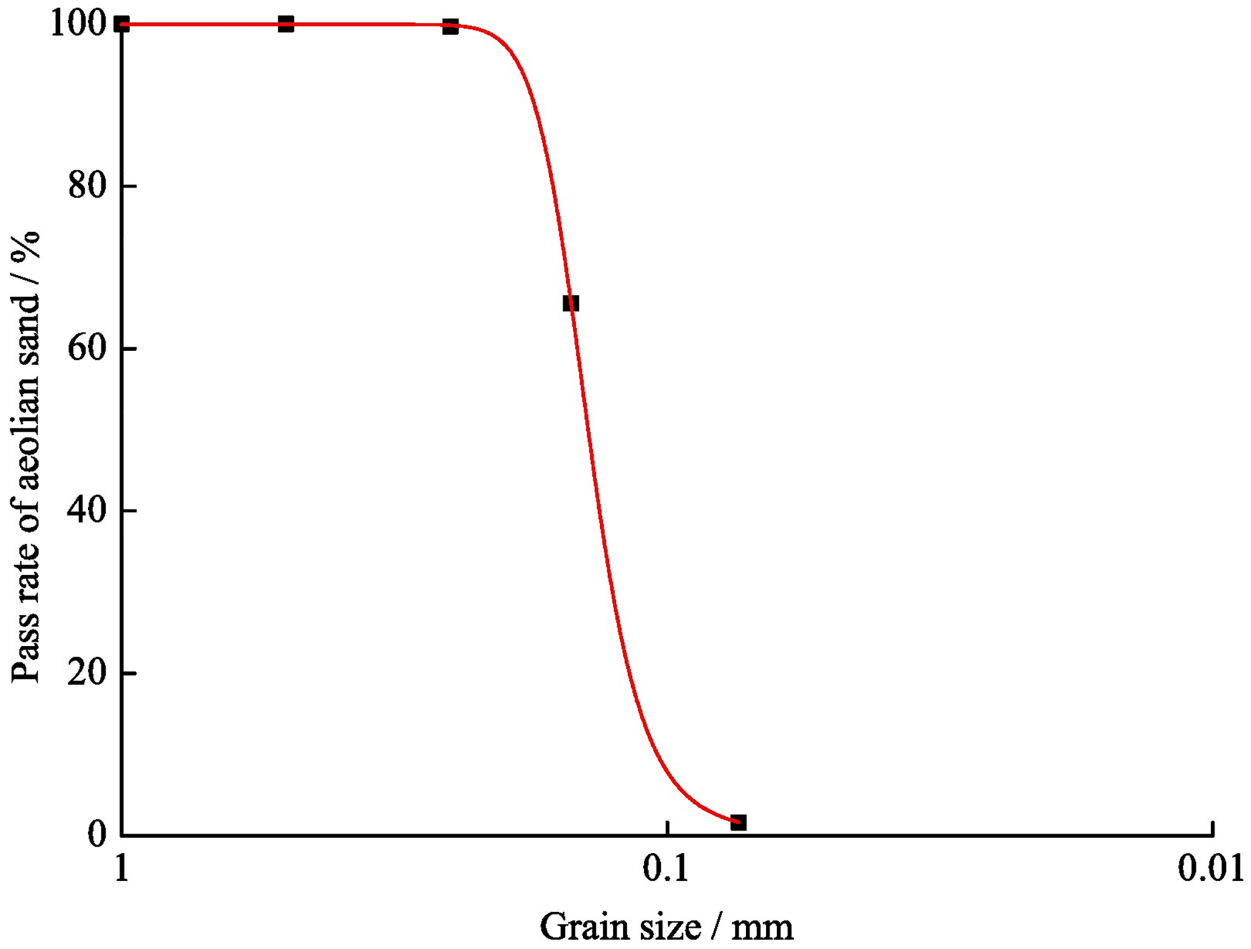
Gradation curves of aeolian sand.

The aeolian sand in this test originated from the Yulin coal mining area in the Northern Shaanxi province, where the aeolian sand has been weathered over a long period. This leads to a loose structure with fine grains and a smooth surface, as well as good water permeability, with a maximum water absorption rate of less than 1%. It is necessary to declare that this mining area does not require specific permission, and field studies do not involve endangered or protected species. In addition, the aeolian sand has few clay particles, and the capillary water height is less than 1 m. The results of granulometric composition by sieving are shown in Table 1, and Fig 3 shows the grain gradation distribution curve.

**Table 1:** Results of aeolian sand sieving test.

Sieve aperture /mm	0.5	0.25	0.15	0.074	Residue
Grading sieve /%	0	0.3	34.1	63.93	1.67
Pass rate /%	100	99.7	65.6	1.67	0

As shown in Fig 3, the grain size of the aeolian sand is mostly distributed between 0.25~0.074 mm (about 60~200 mesh), accounting for more than 90% of the particles. From the calculation of the experimental data, we find that the non-uniform coefficient *C*_u_ is 1.71 and the curvature coefficient *C*_c_ is 0.96. According to the grading criteria, the gravel soil or sand soil can be regarded as a well graded gravel or sand only when *C*_u_ ≥ 5 and *C*_c_ = 1~3. Otherwise, it is considered to be a poor gradation. After comparing and analysing the test results, we conclude that the aeolian sand used in the sieving test is a typical badly graded sand. Therefore, the aeolian sand can be used as a backfill aggregate for backfill mining with loess and fly ash. A proper P.O 42.5# cement can also be added to improve the strength of the backfill body.

In order to better control the overburden strata and surface subsidence and reduce the damage to the surface structures, the packing density of the aeolian sand should be taken into consideration when it is used as a backfill aggregate. Since the packing density is the result of the interaction of different grain sizes, its distribution should be studied. In fact, Andreasen, the principal advocator of classical continuum theory, proposed a new packing theory based on a continuous size distribution [23]. He indicated that the distribution of grain sizes should fit the following equation:

$$P=100{\text{(}D/D_{L}\text{)}}^{\text{n}}$$ (4)

Where *P* is the percentage of grain sizes smaller than size *D* (%), *D*_*L*_ is the largest grain size in the grain system (mm), *D* is the grain size corresponding to *P* (mm), and n is the distribution modulus.

Furthermore, the packing density increases with the decrease in the distribution modulus in the equation, according to the experimental results of Andreasen. However, when the distribution modulus is reduced to 1/3, the closest density is reached, and the distribution modulus is meaningless if it continues to decrease. Based on the sieving test and Eq 4, the distribution modulus *n* was 0.35 when using a grain size of 0.15 mm. We conclude that the porosity between the grain sizes of the aeolian sand in this region is close to the minimum. These grains are mostly in a point-connection structure; therefore, a smaller grain size material can be added to further reduce the voids between the aeolian sand, such as loess (the equivalent grain size is 230~270 mesh) and fly ash (the equivalent grain size is 325~400 mesh). The materials added have large densities and loose structures with quite small compressive capacities due to their structures and improve the backfill body’s strength by using other materials to embed the aeolian sand voids. The materials also improve the mobility, water retention, and cohesiveness, which enhance the pump-ability and impermeability of the mixture. In addition, a material with increasing strength and durability can be formed by reacting with calcium hydroxide or other metal hydroxides in the alkaline loess. At the same time, it is necessary to test the ratio of the backfill materials and slurry concentration to achieve the optimum use of backfill materials.

## Property test of the backfill material

**Fig 4:**
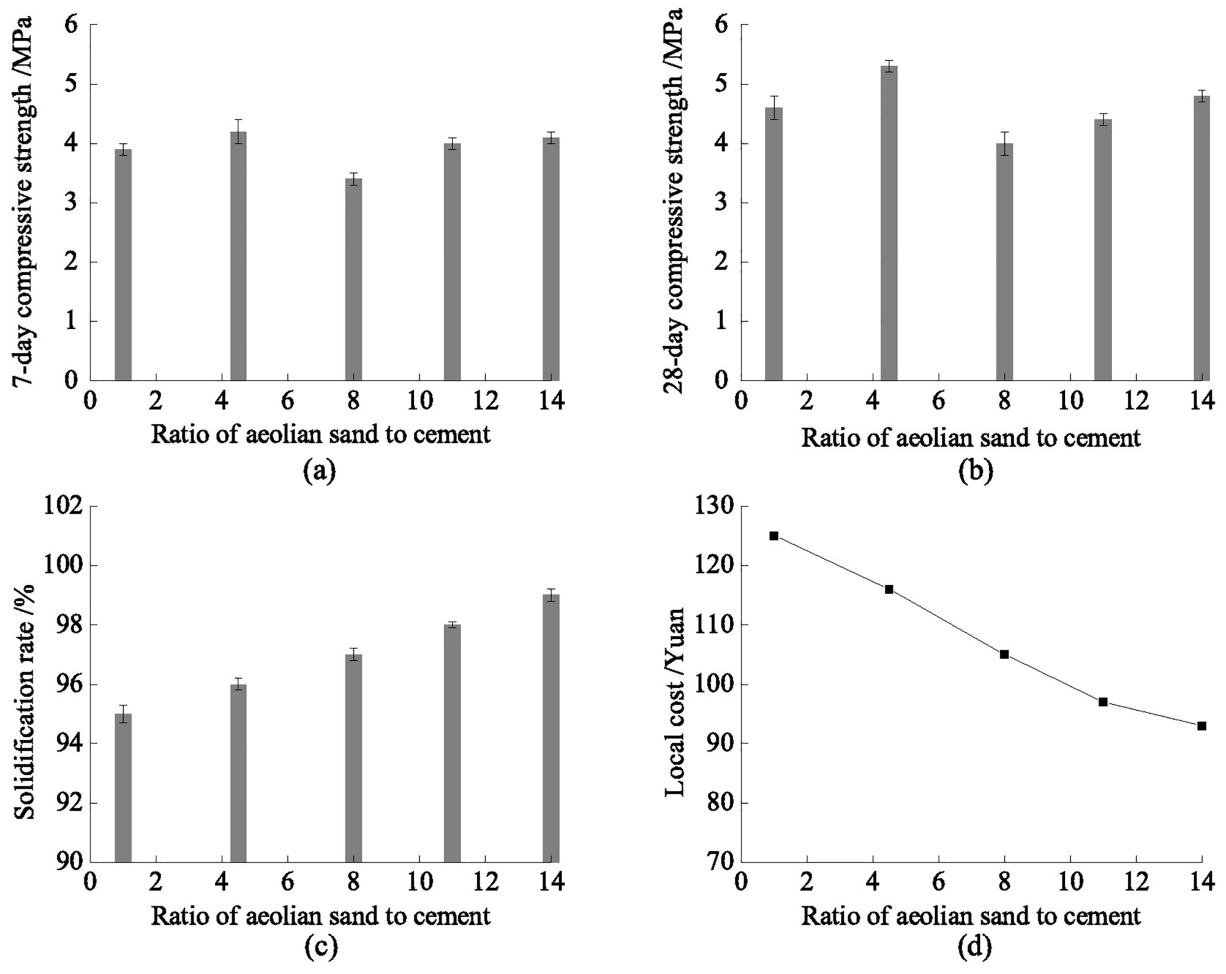
Variation bar for different ratios of aeolian sand to cement.

The backfill material is composed of aeolian sand, which is used as an aggregate, mixed with fly ash, loess, and cement. The fly ash is grade III produced by Jinjie Power Station and the cement is P.O 42.5# cement [24]. Because the fly ash is suspended, the backfill slurry has better mobility and pipeline conveying performance, effectively restraining aggregate deposition. To determine the proportion of backfill material in the experiment, the aeolian sand is regarded as a standard unit for different proportions. According to the experiment, where the backfill material consists of loess and fly ash, the optimal dosage ratio of loess to ash is 1:1.5 ~ 1:3. Considering the porosity of the aeolian sand at 45% and a certain surplus coefficient of 1.2, the ratio of aeolian sand to fly ash is 5:3. Experiments with different ratio of aeolian sand to cement were then carried out using a ratio of aeolian sand, fly ash, and loess of 1:0.6:0.3 and a slurry concentration of 70%. The test block was made by using a cement mortar mould with the dimensions of 70.7 mm × 70.7 mm × 70.7 mm. Each test was repeated three times to ensure accuracy. The variation bar is shown in Fig 4.

From Fig 4, as the dosage of aeolian sand gradually increases, the increasing trend of the backfill body’s strength and solidification rate are not obvious. Considering the local cost, the price of the backfill material decreases gradually. Namely, there is a negative correlation between the dosage of aeolian sand and the backfill cost. Compared with the current backfill materials, the aeolian sand-based backfill materials present a strong mobility and high strength. Combined with the abundant natural aeolian sand, it was determined that the dosage of aeolian sand is 10 times that of the cement. Finally, the ratio of the backfill material is *w* (aeolian sand): *w* (fly ash): *w* (loess): *w* (cement) = 1:0.6:0.3:0.1.

**Fig 5:**
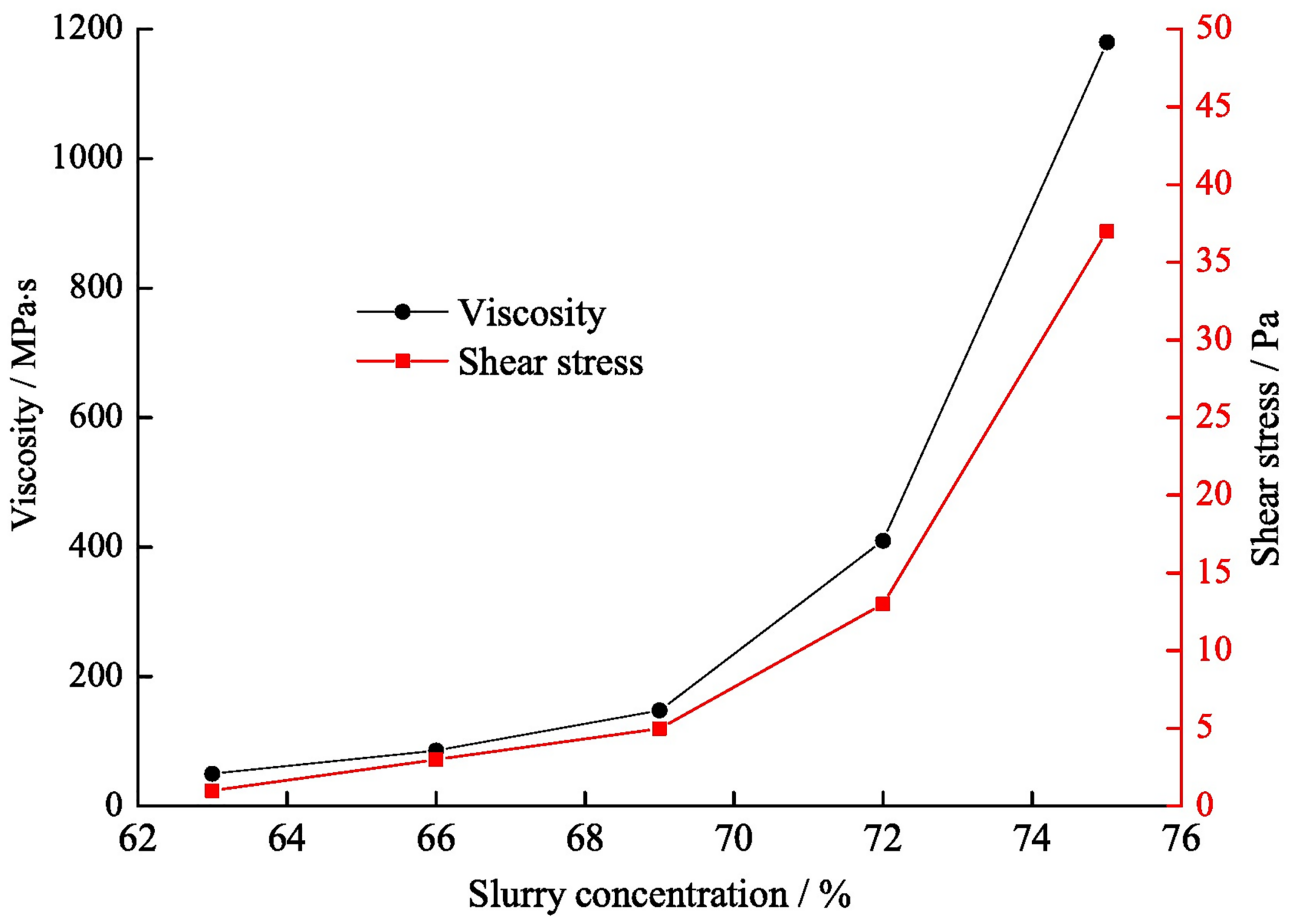
The viscosity and shear stress of slurry at different concentrations.

As the backfill material is transported by the pipeline, the slurry characteristics should be considered in addition to the influencing factors of the pipe itself, mainly including the concentration, viscosity, and slurry temperature. The influence of temperature on the pipeline resistance is achieved by affecting the viscosity. The lower the slurry temperature is, the greater the viscosity. Taking into account the viscosity barely varies at room temperature, the influence of temperature can be neglected. The rheological properties of fly ash slurry are Newtonian, while a high concentration is a Bingham body, and its viscosity is positively correlated with the concentration. Therefore, the optimum slurry concentration should be tested. In determining the backfill material ratio, the influence of the slurry concentration on the rheological properties was studied, and the results are shown in Fig 5 (The data are included in S1 Table).

As shown in Fig 5, with an increase in slurry concentration, the solid content in the unit volume and the interaction degree between grains also increase. This is mainly because the energy required to stabilize the suspended grains increases in the turbulent flow. Therefore, the viscosity and shear stress of the slurry are positively correlated with the concentration. The apparent viscosity and shear stress increased significantly when the slurry concentration exceeded 69%, which indicates that the slurry mobility will decrease markedly when the concentration exceeds 69%. The appropriate slurry concentration can ensure better slurry mobility. Thus, the upper limit of the slurry concentration was determined to be 72%.

## Engineering application

**Fig 6:**
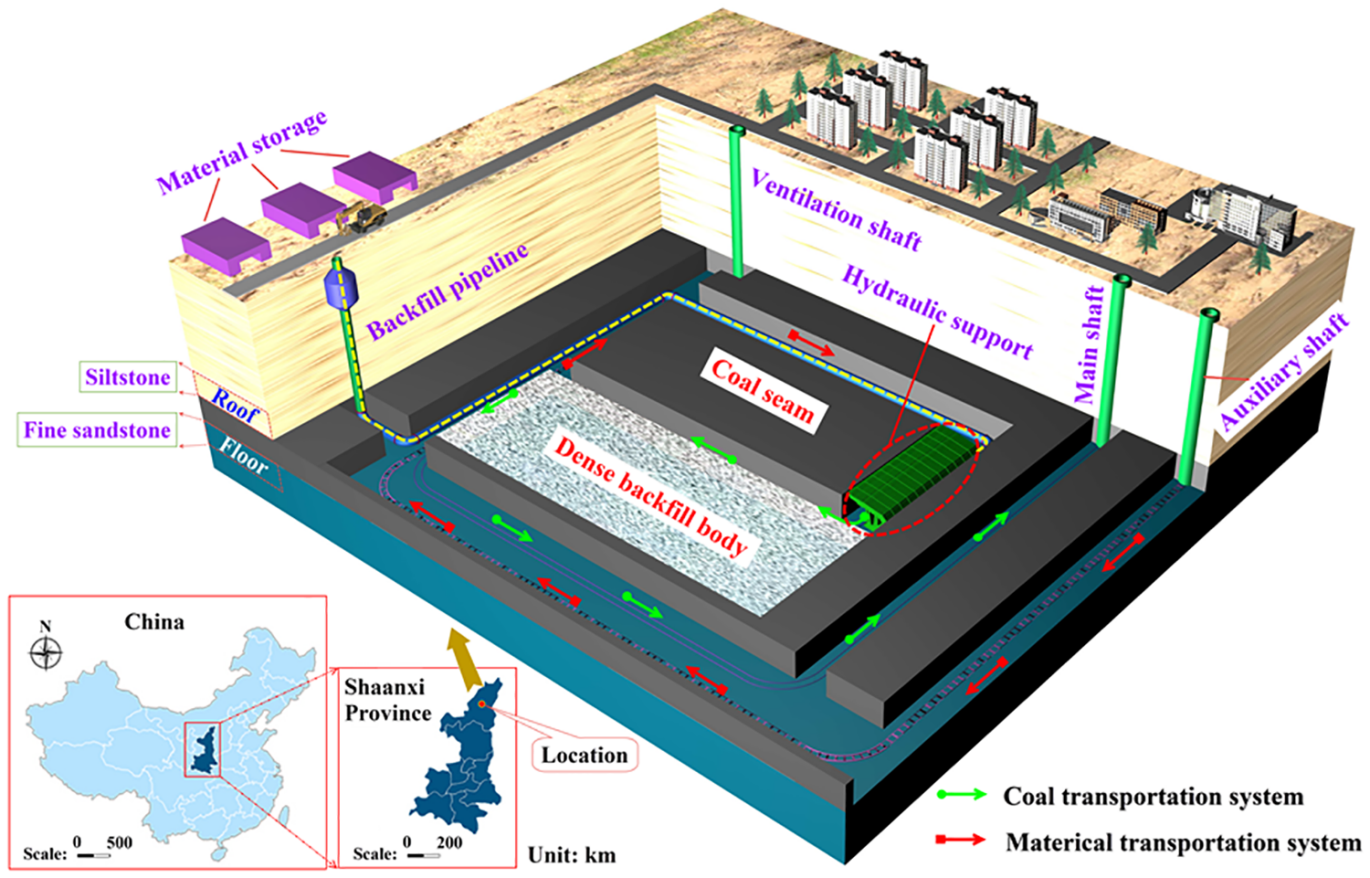
The location of coal mine and route of backfill coal mining.

The Yuyang coal mine (in Yulin District, China), located at the junction of the Maowusu Desert and Loess Plateau, covers a field area of 13 km^2^. This mine has a designed capacity of 3 million t/a. The primary mineable coal is the No. 3 coal seam of the Shanxi group of the Permian system with an average thickness of 3.5 m, which has a stable horizon and simple structure. The buried depth of the seam ranges from 190 to 230 m, and the floor elevation changes between 966 and 971 m. The 2307 working face, located in the No. 23 panel, was selected as the backfill working face. The advancing distance of the 2307 working face is about 1149 m, and the length is 150 m. Based on the engineering design for backfill coal mining, the location of the coal mine and the backfill route are shown in Fig 6.

**Fig 7:**
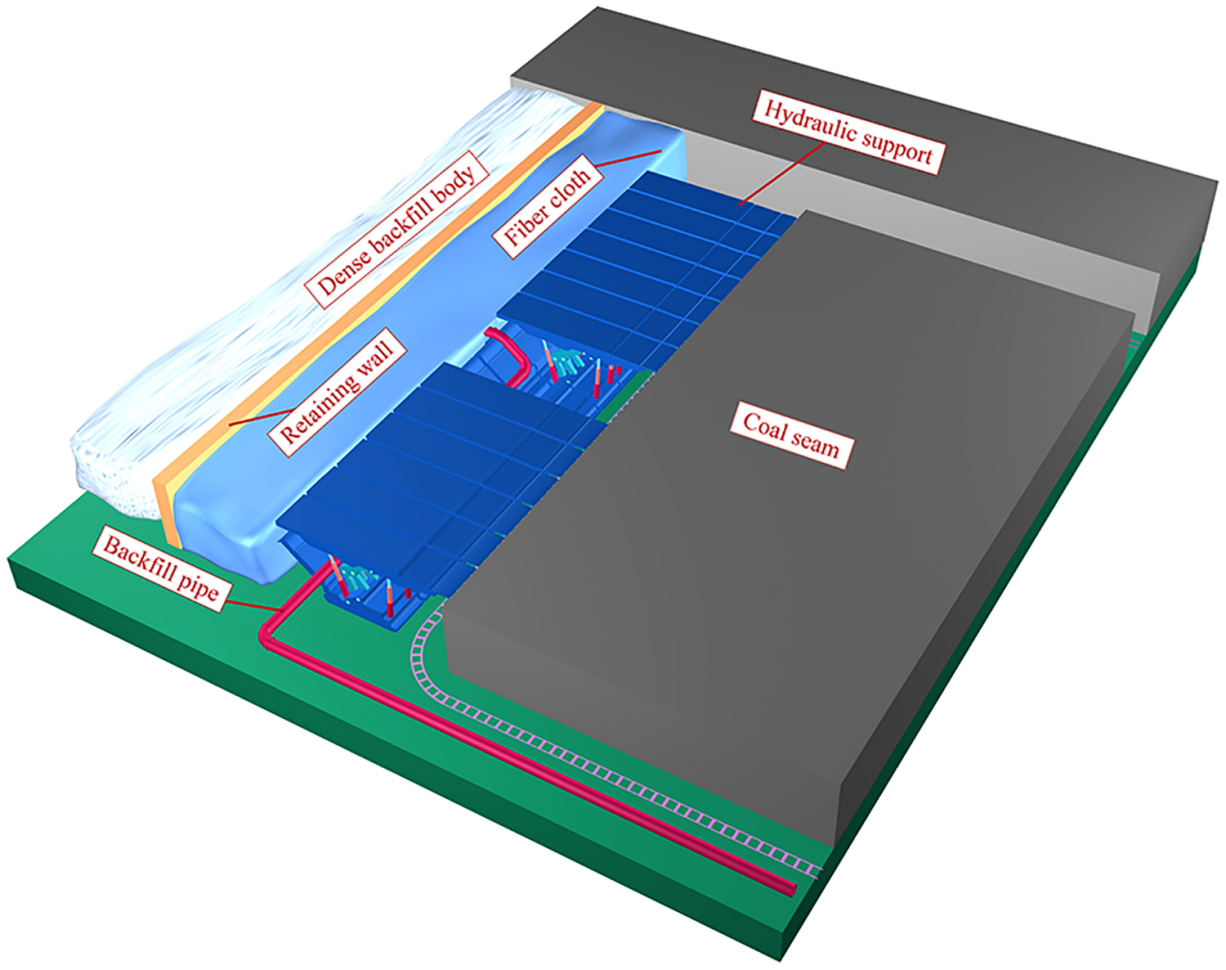
Whole backfill space sealing in the mining face.

**Fig 8:**
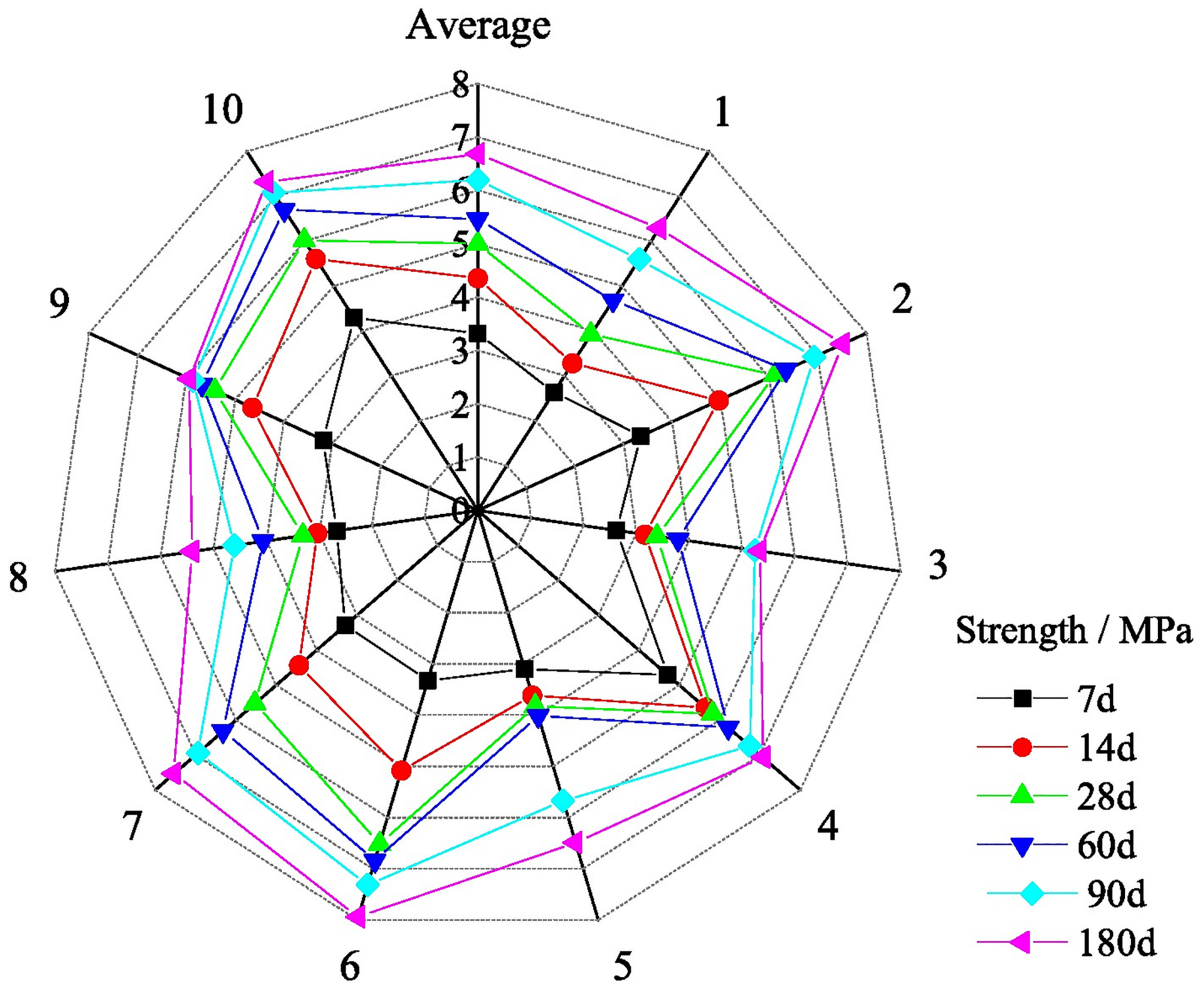
The strength test values of the backfill body.

When backfill mining is carried out on the fully mechanized working face, the backfill step distance was determined to be 6.4 m according to the caved distance of the immediate roof and the safety factor of 1.5 times obtained from similar simulations. The working face is recycled by means of first mining and then backfilling. All the other areas are filled except the tail entry. In order to avoid the influence of slurry on the production of the working face, the whole sealing method is adopted, as shown in Fig 7. Using a pipeline diameter with 14.9 self-flowing lines, the long-distance gravity flow can be carried out without pump pressure [25]. During the solidification of the backfill body, the worker shall renew the fibre cloth for the next cycle. The average backfill rate of the working face was 98% by observation. In different cycles of backfilling in the Yuyang coal mine, samples of the backfill body were collected. The values of compressive strength test are shown in Fig 8.

The results in Fig 8 show that the early compressive strength of the backfill body increases with the passage of time. The average compressive strength increased to 4.35 MPa in 14 days, then the growth rate gradually slowed down, and the long-term strength stabilized above 5 MPa, which is larger than the designed 4 MPa. The strength development and the stability of the ultimate strength can satisfy the pressure of supporting the overlying strata.

**Fig 9:**
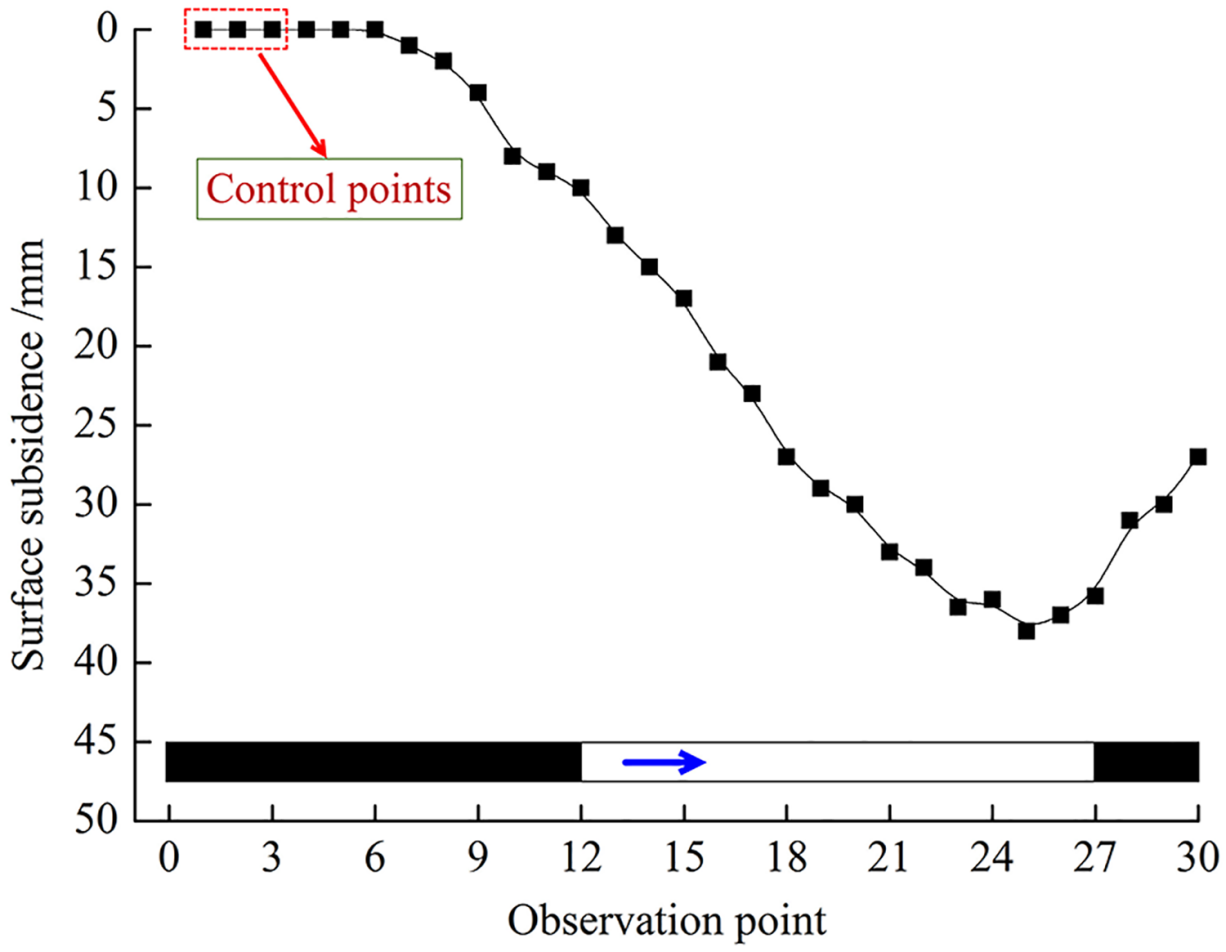
Position of surface subsidence curve and working face.

In addition, the effect of backfilling to control overburden failure can also be reflected from the surface subsidence. Therefore, real-time monitoring of surface subsidence was conducted by using an observation station and an observation line established on the ground. A monthly survey was carried out during the backfilling period. For some reason, the mine was shut down after operating for a year. A total of 72 backfill cycles were carried out in the working face, with a total of 310 m, and 0.21 Mt of coal mined. The strike direction reached critical mining, and the surface subsidence curve is shown in Fig 9 (The data are included in S1 Table).

The measurements indicate that the maximum surface subsidence reached 38 mm. Notably, the maximum horizontal deformation only reached 0.35 mm/m, which is lower than the critical value that would cause damage to buildings. In addition, no obvious changes were observed in the surface building at a horizontal distance of 200 m from the setup room. These results indicate that the material ratio and slurry concentration effectively guarantee overburden failure and surface subsidence.

## Conclusions

Mining operations in northwest China are associated with surface subsidence, water and soil loss, vegetation deterioration, land desertification, and other eco-environmental hazards. The aeolian sand-based backfill material was first used in backfill coal mining to protect the environment and achieve green mining.

Based on the close packing theory, natural aeolian sand in the local mining area has been proven to be an optimal backfill material by the sieving test.

Aeolian sand, loess, fly ash, and cement materials were used to prepare the backfill materials in this study. According to the analysis of the overburden strata movement characteristics after backfilling, under the conditions of a guaranteed backfill rate, a non-uniform coefficient of 1.71, and a curvature coefficient of 0.96, the compaction degree of the backfill body is the key factor in determining the subsidence of the overburden strata. The mechanical properties and proportion of the backfill materials can be determined by testing. Within a certain range, the amount of aeolian sand has a negative correlation with the cost of the backfill material, while the slurry viscosity is positively correlated with the concentration.

The application showed that the maximum surface subsidence was 38 mm and the maximum horizontal deformation was 0.35 mm/m after adopting the backfill coal mining method, which are both controlled within grade I specified by the Criterion of Coal Industry. This indicates that the integrity of the overburden strata is competent. Therefore, this successful practice has proven to be effective in controlling the geo-environment hazards, which is vital for the sustainable development of the mining industry and economic growth.

## Supporting information

S1 Table The data of Figs 5 and 9. (XLS)
